# Complex selective manipulations of thermomagnetic programmable matter

**DOI:** 10.1038/s41598-022-24543-5

**Published:** 2022-12-13

**Authors:** Josu Irisarri, Iñigo Ezcurdia, Xabier Sandua, Itziar Galarreta-Rodriguez, Jose Ignacio Pérez-Landazabal, Asier Marzo

**Affiliations:** 1 UPNA ISC, UpnaLab, 31006 Pamplona, Spain; 2 Physics Department, UPNA, 31006 Pamplona, Spain; 3 Science Department, UPNA INAMAT2, 31006 Pamplona, Spain

**Keywords:** Engineering, Materials science, Optics and photonics

## Abstract

Programmable matter can change its shape, stiffness or other physical properties upon command. Previous work has shown contactless optically controlled matter or magnetic actuation, but the former is limited in strength and the latter in spatial resolution. Here, we show an unprecedented level of control combining light patterns and magnetic fields. A mixture of thermoplastic and ferromagnetic powder is heated up at specific locations that become malleable and are attracted by magnetic fields. These heated areas solidify on cool down, and the process can be repeated. We show complex control of 3D slabs, 2D sheets, and 1D filaments with applications in tactile displays and object manipulation. Due to the low transition temperature and the possibility of using microwave heating, the compound can be manipulated in air, water, or inside biological tissue having the potential to revolutionize biomedical devices, robotics or display technologies.

## Introduction

Programmable matter can change shape, density, moduli or other physical properties in a programmatic way^1^. These changes are controlled externally or triggered by embedded sensing and processing in the material^2^. The main two approaches towards implementing programmable matter are: modular robots^3^, which provide more intelligence; and external actuation^4^, which yields a higher spatial resolution and scalability. Programmable matter holds ground-breaking applications in engineering and medical fields, but the granularity that can be achieved in its manipulations is still significantly limited.

Light has been used as an external actuation method. Materials combined with azobencenes^5^ are actuated when illuminated. For example, triggering movement when a reflective or opaque object approaches the material^6^, or enabling locomotion on filaments and cylinders when illuminated with dynamic light patterns^7^. On the other hand, the heat generated by light can move small objects on the water surface due to temperature gradients^8^ or change phase in shape-memory alloys^9^. Actuation with light or its thermal effect has high spatial resolution given the existing technology to project images, yet the actuation strength is relatively weak and after actuation, the whole material returns to its initial state or retains a non-reversible state. Moreover, light cannot pass through opaque materials.

Magnetic fields are another way of controlling matter from a distance. A flexible thread of polymer containing magnetic powder can be steered remotely to navigate contorted environments^10^, sheets of flexible materials embedded with ferromagnetic or magnetic particles can be translated and flexed in a controlled way for locomotion^11,12^, a carpet made of magnetic cilia can be actuated to control the objects that are on top of it^13^, and magnetic slime can be moved magnetically to trap and transport other objects^14^. Magnetic actuation is strong and can pass through non-metallic materials yet it is not possible to have high-spatial resolution since magnetic fields do not remain focused at a distance. For better control, the magnetic attraction or repulsion on the material can be modulated by heating it up towards its Curie temperature, either using light^15^ or electromagnetic induction^16^, yet these methods are applied on the whole surface not allowing fine manipulation. Liquid metal can be translated in droplets^17^ by external magnetic fields, and when they are combined in a magnetorheological slurry it can also change stiffness^18^, serving as dynamic electrical connection in reconfigurable circuits.

Here, we show unprecedented levels of control manipulating matter using a combination of thermal spatial patterns and magnetic actuation on a composite material made of a matrix of low-temperature reversible thermoplastic (Polycaprolactone, PCL) mixed with ferromagnetic powder (iron particles), see “Methods” “Compound mixing”.

Thermoplastics have been mixed before with iron powder for tuning its thermal conductivity^19^, electrical resistance^20^ or oxygen absorption^21^. In the context of programmable matter, the combination of magnetic and thermal actuation has been shown for this kind of compound^22,23^, yet the manipulations were only applied to 2D sheets and the heat was applied to the whole material, limiting significantly the types and complexity of the manipulations.

**Figure 1:**
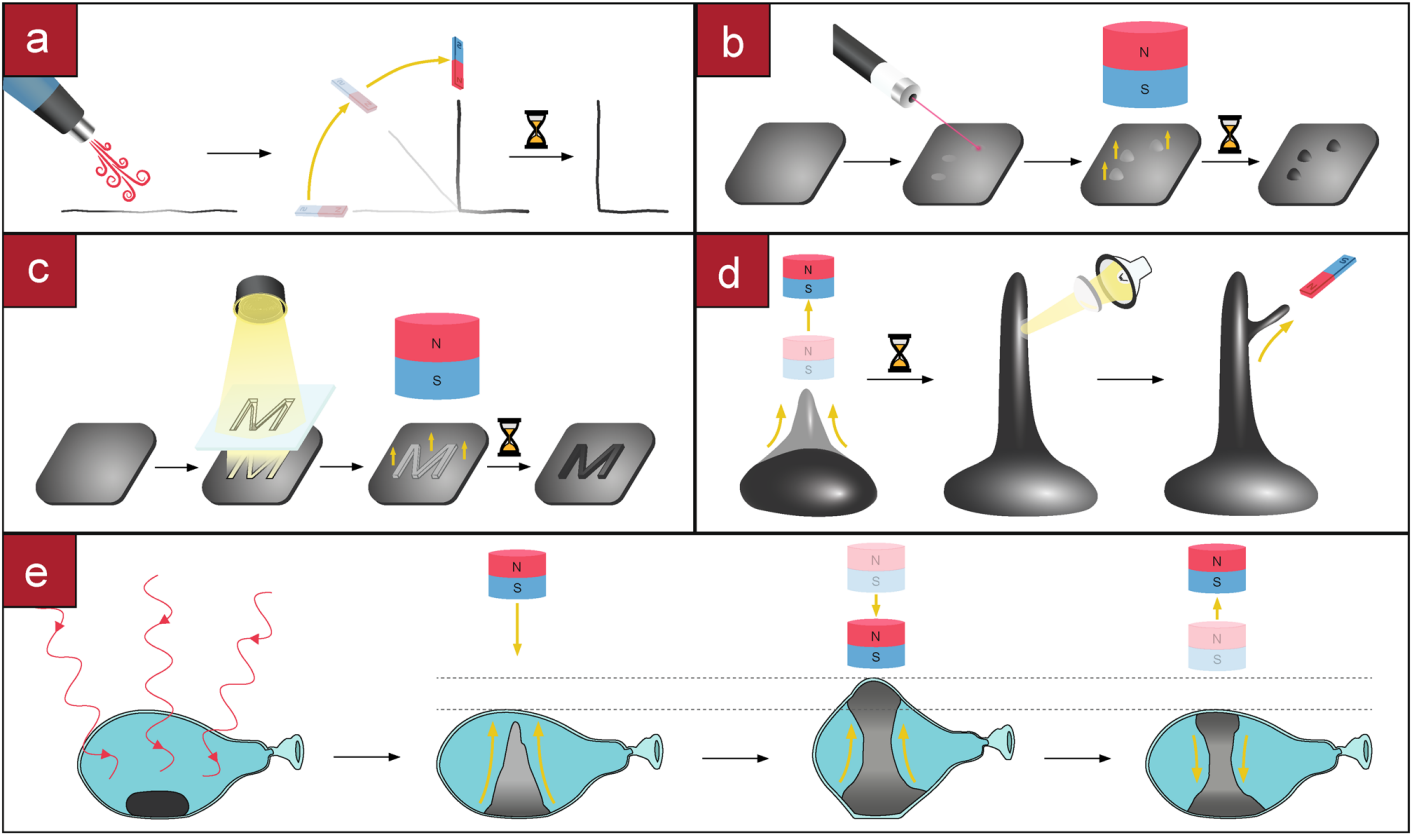
Manipulations on a ferromagnetic thermoplastic using thermal patterns and magnetic fields. **(a)** Hot air makes the centre of a filament malleable, a magnetic field pulls one side of the filament that bends along the heated area, on cool down the filament is solidified. **(b)** A laser heats up specific points on a sheet, a magnetic field attracts those points upwards, on cool down the raised points are fixed and can be pressed without deformation. **(c)** A 2D pattern is illuminated into a sheet of material, when a magnetic field is applied, the heated areas rise and on solidification they become embossed. **(d)** A blob of material rises into a column, a point on the column is heated and a secondary branch is pulled from it. **(e)** Microwaves can heat the material when it is inside an optically opaque material for example to expand its container or actuate it.

In our presented manipulations, the material is solid at room temperature (25 ${^\circ }$C) but becomes malleable beyond 50 ${^\circ }$C by the application of heat on specific locations. The heat can be applied globally, with an IR lamp; at a localized area, utilizing hot air guns; with spatial patterns, using a mask on collimated light; at a focal spot, using focused halogen lamps or lasers; and inside opaque materials, with microwave radiation (see “Methods”: “Thermal sources”). Then, a magnetic field attracts the Fe particles that are embedded within the matrix, dragging the malleable parts of the PCL with them. The material solidifies when it cools down at room temperature, being this process repeatable. Different applications ranging from the formation of Braille code to sculpting figures are enabled using spatially complex thermal patterns applied on 1D filaments, 2D sheets or 3D blocks (see Supplementary Movie 1). The basic principle is shown in Fig. 1.

A qualitative model of the working principle is shown in Supplementary Movie 2. The following equations were combined in a connected particle time-domain simulation to inform the experiments presented in the paper.The H-Field generated by the magnet is approximated as a dipole^24^: $\textbf{H}(\textbf{r}) = \frac{1}{4\pi }\left( \frac{3 \hat{\textbf{r}}(\textbf{m} \cdot \hat{\textbf{r}})-\textbf{m}}{r^{3}}\right)$ where $\textbf{r}$ is the vector from the dipole to the point in the field, and $\textbf{m}$ is the magnetic moment of the dipole. This magnetic force that the H-field exerts on an iron particle is calculated as^25^: ${\rm{F}}_{\rm{m}}=\mu _{0} {\rm {~V}}_{\rm{p}} {\textbf{M}}_{\rm{p}} \nabla \left( {\textbf{H}}_{\rm{p}}\right)$ where $\mu _{0}$ is the Vacuum magnetic permeability,${\rm {~V}}_{\rm{p}}$ is the particle volume and ${\textbf{M}}_{\rm{p}}$ is the magnetization of the particle calculated as^25^. The distribution of the heat inside the material is modelled using the common heat equation $\frac{\partial u}{\partial t}=\alpha \Delta u$ where $\alpha$ is the thermal conductivity. The viscosity of the material determines its shear displacement for a given force $F=\mu A \frac{u}{y}$ where $\mu$ is the viscosity, *A* is the area, and $\frac{u}{y}$ is the rate of shear deformation; for a thermoplastic, its viscosity ($\mu$) decreases logarithmically with temperature^26^.

## Results

### Characterization of the material

Multiple samples with different proportions of iron particles were created and moulded into cylinders (see “Methods”: “Compound mixing”), mechanical and magnetic characteristics were measured. The samples were labelled from S10 (10% of iron in volume) to S50 (50% iron volume), adding iron powder beyond 50% volume made the sample brittle and not able to maintain its structure.

**Figure 2:**
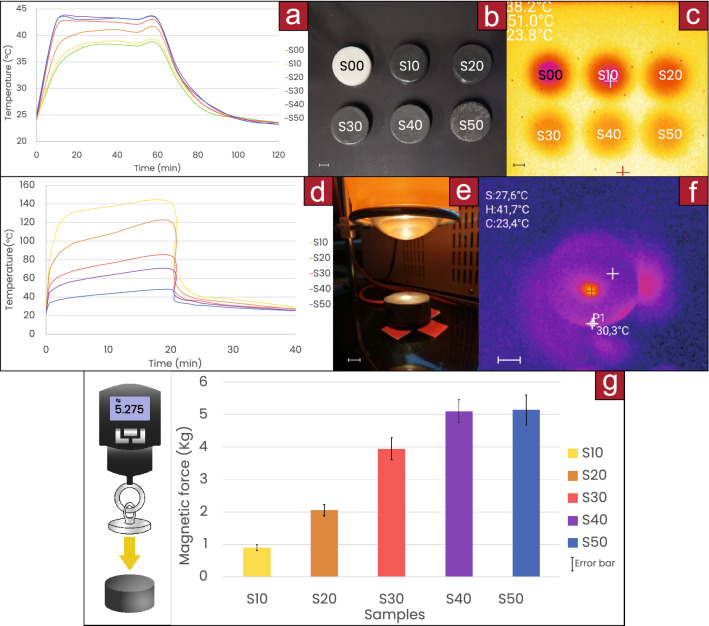
**(a)** Temperature on the top of the samples over time when placed on a heated bed at 50 ${^\circ }$C, the heat is removed after 60 min; with color **(b,c)** thermal images. **(d)** Temperature over time at the side of the samples when a focal spot is applied at the center, the heat is removed after 20 min; with color **(e,f)** thermal images. **(g)** Magnetic pulling force on the samples.

Thermal conductivity was monitored through the cylinders of 5 cm diameter and 3 cm thickness (Fig. 2a) when they were placed on a closed-loop heated bed at 50 ${^\circ }$C (Fig. 2b,c). The larger the iron content, the greater the thermal conductivity through the material, temperature was measured on the top surface of the cylinders. Surface conductivity was also monitored (Fig. 2d–f), the lower the content of iron, the higher the temperature reached and the longer it took to cool down; this may happen because the heat cannot be distributed through the material. More details are in “Methods”: “Thermal measurements”. The light intensity can be regulated to raise the temperature of the material at different rates (see Supplementary Image 7).

Magnetic pulling forces were measured on the different samples and are shown in Fig. 2g. The larger the iron content, the stronger the magnetic force. The maximum magnetic attraction distance was similar for all the samples ($\approx$6 cm) since samples with more iron volume were more strongly attracted but also weighted more. More details are in “Methods”: “Magnetic measurements”. Additional characterization (Hardness Shore and Tensile Tests as well as Microscopic, confocal and SEM images) are available in Supplementary Figs. 8, 9, 10, 11, 12 and 13 respectively.

The material was homogeneous in colour after preparation and actuation. Furthermore, the density of different parts showed a very small deviation (SD $=$ 0.006 g/ml) as is shown in Supplementary Image 6. This indicates that the powder had a homogenous distribution even after actuation and that no agglomeration of powder occurred after the magnetic field actuation.

### Basic manipulations

Basic operations on the material are performed by heating up a specific area, attracting that area with a magnetic field and then cooling it down by passive dissipation or actively with a cold air jet. The material can be employed in different dimensionalities: 1D filament, 2D sheets and 3D slabs.

**Figure 3:**
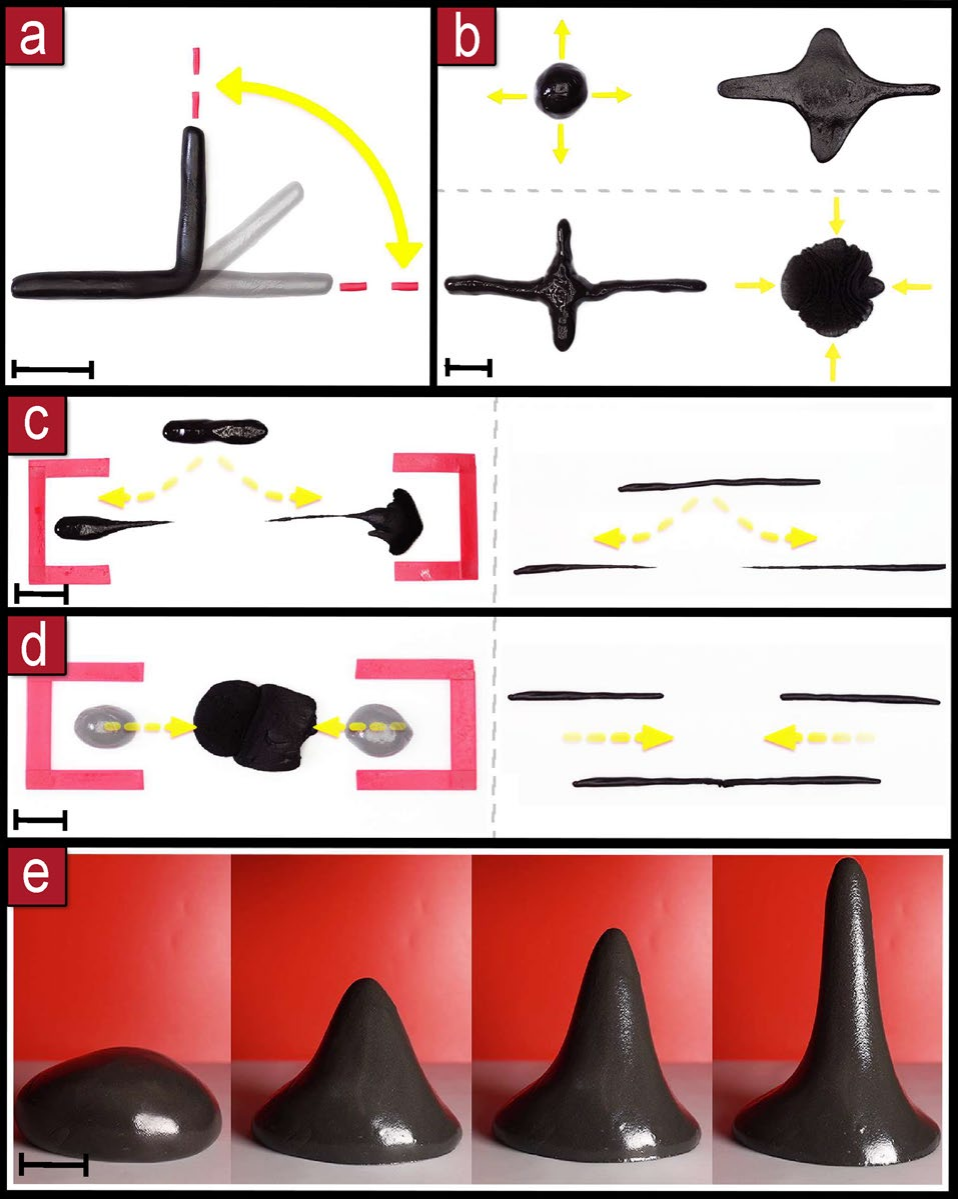
Basic manipulations. **(a)** Bending and unbending. **b)** Stretching and contracting. **(c)** Splitting. **(d)** Merging. **(e)** Rising. Scale bars are 3 cm.

When the material is at solid state, a magnetic field can translate it along surfaces using regular magnetic attractive manipulation. Since we used ferromagnetic Fe particles, the compound is always attracted by the magnet. Precise and selective control is possible. Using two magnets, rotation of elongated pieces of material was achieved. Applying heat can melt and flatten specific parts of the material or the whole piece if applied globally either with magnetic forces pulling from below or by the action of gravity. These basic manipulations are shown in Supplementary Fig. 5.

Bending of elongated pieces can be achieved at a targeted location by applying focused heat on the desired pivot point, then applying a magnetic field above the pivot; the other part can be held in placed: by its own weight if it is large enough, by another magnet or by sticking it with heat; unbending operations are performed with the reverse process (Fig. 3a). Stretching and contraction is accomplished when both parts (above and below the heated area) are moved by separating or approximating them respectively (Fig. 3b). If the solid parts are separated further, the heated malleable part breaks and the material splits into two parts (Fig. 3c). Joining separated pieces can be performed by heating them on the target joining areas, and then pushing these areas together (Fig. 3d). Rising of a part can be achieved by heating up the whole sample and then applying a magnetic field from above (Fig. 3e).

Heating the surface of the material can be achieved in 1 min with light, heating the whole piece can be done in 10 min. Bending was performed at 5$^\circ$/s, the stretching and contracting manipulation took 10 s, the duration was similar for splitting. To achieve full separation into two parts, a minimum distance of 5 cm. was required. The rising speed was 5 mm/s, it is possible to go faster but precise control is needed to avoid that the material gets into the magnet. The maximum height was 10 cm, longer branches can be obtained but they needed to be cooled down externally to avoid collapse.

### Complex manipulations

**Figure 4:**
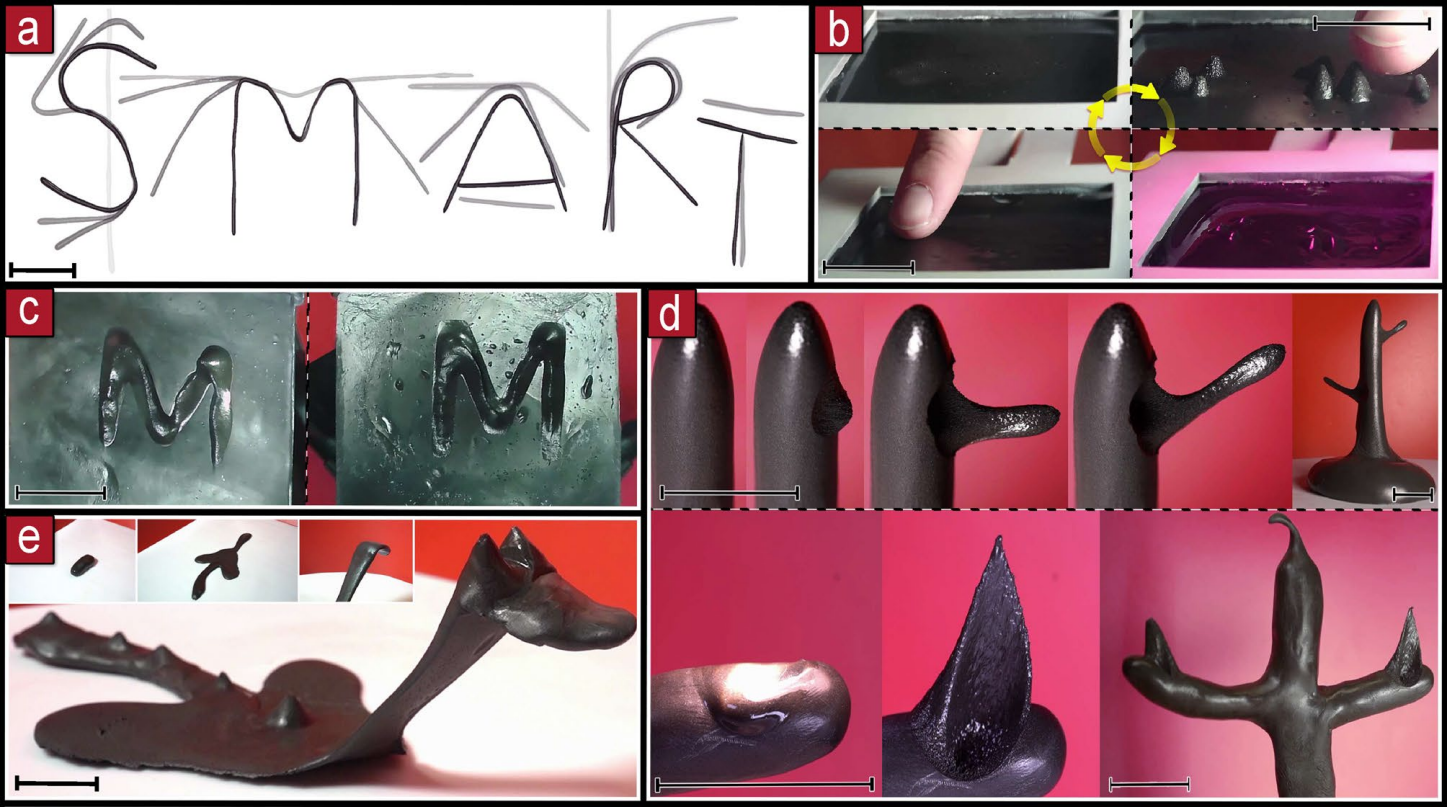
Complex manipulation. **(a)** Forming letters with 1D filament. **(b)** Reversible Braille patterns on a sheet. **(c)** Embossed letter M on a sheet. **(d)** Branching. **(e)** Sculpting in 3D. Scale bars are 3 cm.

Different letters can be created starting from a single strand of filament by combining basic operations. The filament is split into the different segments that will make each letter. The separated parts were bent at targeted positions at specific angles. Then, some segments were joined together. Each letter can be moved as a whole solid afterwards. The letters, S,M,A,R,T were created using this process (Fig. 4a).

Arbitrary patterns can be raised in a sheet by applying light patterns to make it malleable at specific positions. Then, a magnetic field from above, rises the malleable areas. Braille patterns (Fig. 4b) and the contour of the letter M (Fig. 4c) were embossed on sheets. This process is reversible by applying heat on the whole surface for flattening it out.

An organic tree shape was created by rising a blob of material into a main trunk, then sub-branches were extruded (Fig. 4d). A sculpture of a sea creature was created from a lump of material. Firstly, it was flattened by melting. Then the general shape of the tail, neck and fins was stretched. Secondly, spikes were risen along the tail. Thirdly, the low-neck was heated and then raised by pulling from the top. Finally, the end part of the neck was moulded into a head by bending it down and raising two small antennas (Fig. 4e), we note that all these steps were done from a distance.

### Use cases

**Figure 5:**
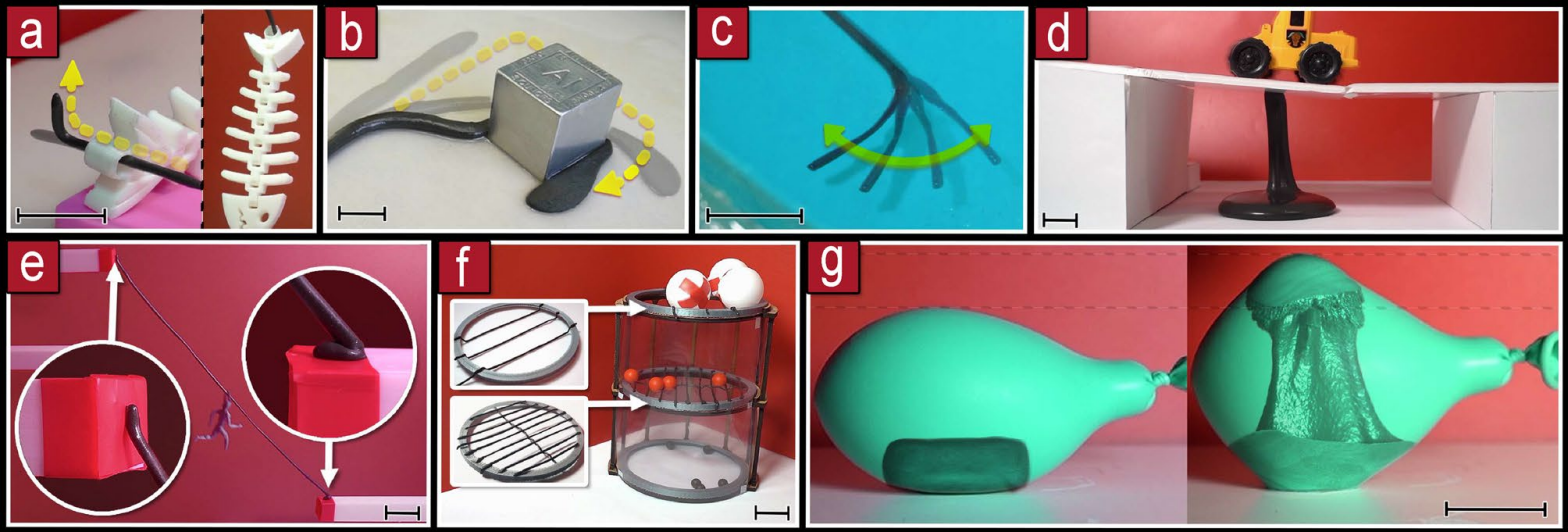
Use cases. **(a)** A hook to grab a small object. **(b)** A larger hook to drag a heavier object, can pull up to 20 kg. **(c)** Operation under water. **(d)** Lifted column as a support structure. **(e)** Filament sticking to separated places to connect them. **(f)** Filaments stacked at different distances for selective filtering. **(g)** Heating of the material inside a container using microwaves for being able to expand and contract it dynamically, after solidification it maintains the container expanded. Scale bars are 3 cm.

Flexible filaments doped with magnetic powder can be used to navigate through contorted environments^10^, here we can also control which parts of the filament become flexible or rigid by heating them up, this enables to use the filament as a hook to grab objects in air (Fig. 5a,b) or water (Fig. 5c).

The mechanical properties of the thermoplastic enables the material to work as a support for structures (Fig. 5d). When the material is heated up beyond 80 ${^\circ }$C and then cooled down it sticks to different surfaces, this can be used to connect separated areas (Fig. 5e) and filter passing objects (Fig. 5f).

The material can be heated up also when it is inside optically-opaque media using microwaves. A 2.45 Ghz magnetron heated up the material making it malleable while it was inside a lung simulator balloon. Then, with a magnetic field it could be expanded and shrunk in a controlled way, when the material solidified, it kept the balloon expanded (Fig. 5g).

## Discussion

The magnetic fields have a limitation on their reach of $\approx$6 cm, yet the employed magnets were regular neodymium cylinders. Stronger magnetic fields from specialised equipment would provide a larger reach.

Heat diffusion is necessary for making the material malleable beyond the surface where the light illuminates the material. However, diffusion makes the thermal patterns less sharp and limits the achievable steepness on surface deformations. Materials with less concentration of iron have less conductivity through the material and get hotter on the surface, thus are better suited for surface manipulations like Braille codes. Compounds with more iron are more adequate for manipulations on the bulk (e.g., bending, splitting, rising or melting). Microwave heating can be used for bulk heating even if thermal conductivity is low because it penetrates on the material, whereas light-based methods or air are more suited for surface heating.

Homogeneous compounds were obtained by mixing the thermoplastic polymer with iron particles. Given the size of the particles and viscosity of the material, the compound retained its uniformity after multiple manipulations (i.e, the particles dragged the plastic with them) on the same piece. Oxidation of the iron particles or change of colour was not observed in the materials throughout the experiments, even on the samples that were immersed in water.

The magnetic fields where controlled in a close-loop manner by moving and rotating the magnets according to the visual observation of the material reactions. However, heating was applied in open-loop, more control could be obtained in the thermal patterns if they were applied in close-loop with a thermal camera.

The employed thermoplastic (PCL) is biocompatible, iron particles (or their oxide) are of common use in medical applications. Additionally, microwave radiation can heat up iron particles without heating up significantly the surrounding biological tissue^27^. This makes the compound viable for operations inside the human body manipulating it from the outside, for example along the digestive tube or upper respiratory tract.

In this paper, we focused on the mechanical manipulations that are only possible due to the thermomagnetic actuation, different functionalizations can be added to the material. For example, adding silver compounds to make it conductive and functional in reconfigurable circuits, or antibiotic microcapsules at the centre that get released when the material is split.

Remote manipulations of unprecedented complexity have been shown controlling a ferromagnetic thermoplastic with thermal patterns and magnetic fields. This works opens up applications in tactile displays and object manipulation. We highlight the capability of microwave and magnetic fields to pass through optically opaque materials, making these manipulations suitable for operating in biological tissue or plastic containers.

## Methods

### Compound mixing

The samples were prepared by solution casting a mixture of polycaprolactone (PCL, Polydoh Materialix) with iron particles of 100$\mu$m with 98% purity (Alquera). Dichloromethane (DCM) and hexane (Sigma Aldrich) solvents were used to ensure good blending.

In this process, five samples (S10, S20, S30, S40 and S50) with different iron content in volume were prepared inside a cubicle with an extractor hood and under normal conditions at room temperature. A beaker of 1L capacity was placed on a hotplate IKA C-MAGHS7 with a temperature controller IKA ETS-D5 and a digital stirrer LaboLan OS40-S, equipped with a mixer paddle. First, the DCM solvent temperature is settled at 30 $^{\circ }$C inside the beaker. The amount of solvent was ten times in volume the mass of polymer to be dissolved, e.g. 200 ml solvent for 22 g of PCL. Then, the polymer was added. The reaction was carried out at $30{\pm }2 \,^{\circ }$C and stirred for 2 h at a speed of 300 rpm. Once the polymer was totally dissolved, the iron powder was incorporated to the solution and mixed for 1 h, increasing the mechanical stirring speed up to 500 rpm. After the iron particles were completely dispersed, 100 mL of hexane was added to promote the composite precipitation and the solution temperature was increased to $50{\pm }2 \, ^{\circ }$C in order to evaporate both solvents.

Once the mixture is homogeneous and the solvents have disappeared, the mixture can be removed and put into a mould. For minimising the differences within the experiments, a volume around 21.34 cm$^{3}$ was settled for each sample considering the mould capacity and materials density: PCL ($\rho =$ 1145 g/cm$^3$) and iron ($\rho =$ 7874 g/cm$^3$). These silicone moulds were used for creating 3D cylindrical shape samples. The 2D sheets were crafted the same way, but instead of putting them in a mould, they were flattened with a stainless steel rolling pin on a silicone mat. For the 1D filaments, the compound was shredded into pellets and introduced in an extruder (FelFil Evo equipped with a 1.75 mm circular cross section nozzle) at 60 $^{\circ }$C and 6 mm/s of speed. Samples can be seen in Supplementary Fig. 1 and the resulting weight of the volume proportions in Table 1.

**Table 1:** Iron and PCL proportions in volume and weight for each sample.

Samples	S10	S20	S30	S40	S50
Fe volume (%)	10	20	30	40	50
PCL volume (%)	90	80	70	60	50
Fe (g)	16.81	33.61	50.42	67.21	84.03
PCL (g)	22	19.5	17.11	14.66	12.22

### Thermal sources

**Figure 6:**
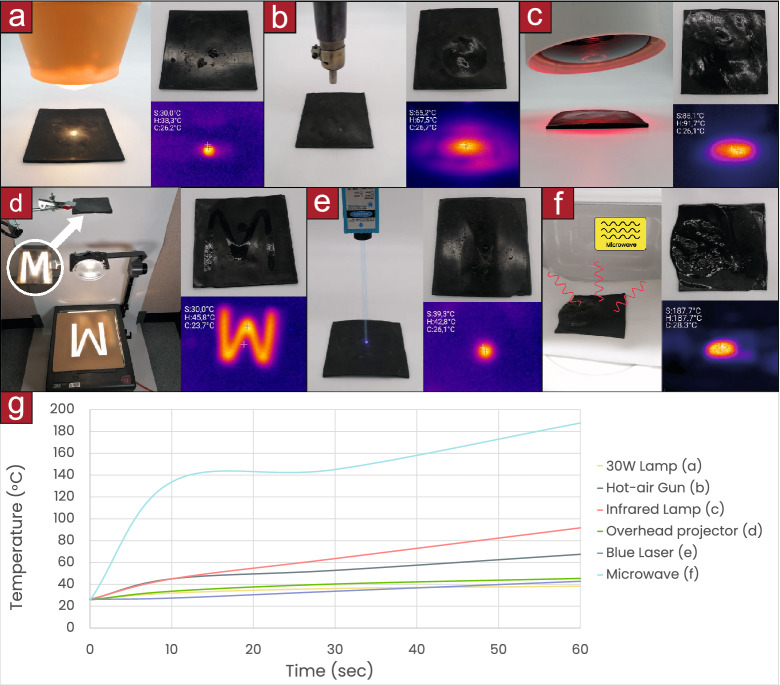
**(a)** Heating with a GE 4405 PAR36 lamp of 12.8 V and 30 W focusing the light with a planoconvex aspheric lens LAF6075. **(b)** Heating with a MMOBIEL Yihua 858D hot-air soldering station. **(c)** Heating with Beurer IL21 infrared lamp. **(d)** Heating with a 3M 9000 AHKS overhead projector: using a mask and a focusing planoconvex aspheric acrylic lens of 90 mm $\varnothing$ and F $=$ 20 mm. **(e)** Heating with a Taurus W750MG microwave. **(f)** Heating with a Vevor 3B 450 nm and 2500 mW blue laser. **(g)** Temperature evolution using different heat sources over 60 s.

Different thermal sources were used during the experiments: a focused halogen lamp, hot air gun, IR lamp, acetate masks projector, laser and microwave. They are shown in Fig. 6.

### Thermal measurements

A thermal camera RS PRO T-10 (RS Components) was used to measure the temperature on the samples at different points. In the bulk thermal conductivity experiment, the samples were heated on a hot-bed from an (Ender 3 PRO 3D printer) that was set in closed-loop at 50 ${^\circ }$C. In the point spread thermal experiment, the focused halogen lamp (GE 4405 PAR36 30 W) was used to heat the middle point of the surface of each sample. The focal light was set at a distance of 5cm from the tip of an aspheric acrylic lens (Knight Optical LAF6075). The lamp was working at its nominal power.

### Magnetic sources

Different magnets were used for generating the magnetic fields that manipulated the samples they can be seen in Supplementary Fig. 3. Their field was measured with a teslameter (WT10A). The magnets were moved manually, with tripods or with linear stages.

### Magnetic measurements

For the magnetic force measurement, each sample was attached into a magnet (Wukong 304 M8 neodymium N52) and an increasing downwards force was applied until the sample detached from it. The magnet was hanged from a digital dinamometer (GPISEN 50 kg, 5 g error) as it is shown in the Fig. 6g. The force was recorded as the maximum force needed to detach the magnet.

## Supplementary Information

Supplementary Figures.

Supplementary Video 1.

Supplementary Video 2.

Supplementary Video 3.

## Data Availability

Data generated or analysed for this research are included in the published article and its supplementary information files.
