# Supplementary material for: Complex selective manipulations of thermomagnetic programmable matter
Source: Sci Rep. 2022 Dec 13;12:20767. doi: 10.1038/s41598-022-24543-5 (PMC9747806; doi:10.1038/s41598-022-24543-5)
Supplement: Supplementary file 1 — Supplementary Figures. [file 41598_2022_24543_MOESM1_ESM.pdf]

Supplementary Information: Complex Selective Manipulations of Thermomagnetic Programmable Matter

Josu Irisarri[1], Iñigo Ezcurdia[1], Xabier Sandua[2], Itziar Galarreta-Rodriguez[3], Jose Ignacio Pérez-Landazabal[3], and Asier Marzo[1,*]

[1]UPNA ISC, UpnaLab, Pamplona, 31006, Spain
[2]UPNA, Physics Department, Pamplona, 31006, Spain
[3]UPNA INAMAT2, Science Department, Pamplona, 31006, Spain
[*]asier.marzo@unavarra.es

ABSTRACT

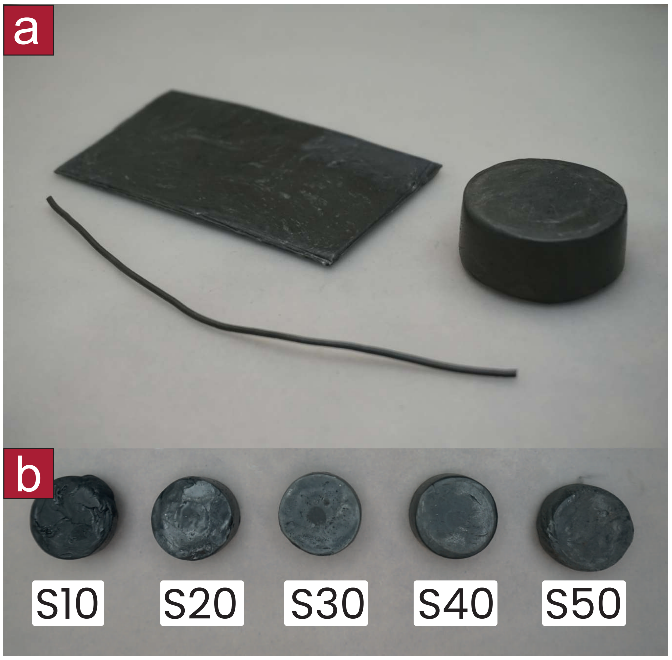

Figure 1. a) Samples of filament of 1.75 mm diameter, 2D sheet of 2 mm thickness and 3D cylinders of 40mm of diameter and 20 mm thickness. **b)** Cylinder samples with different proportions of iron (S10 = 10% iron in volume, S50=50% iron in volume). The labelled number indicates its content of iron in volume.

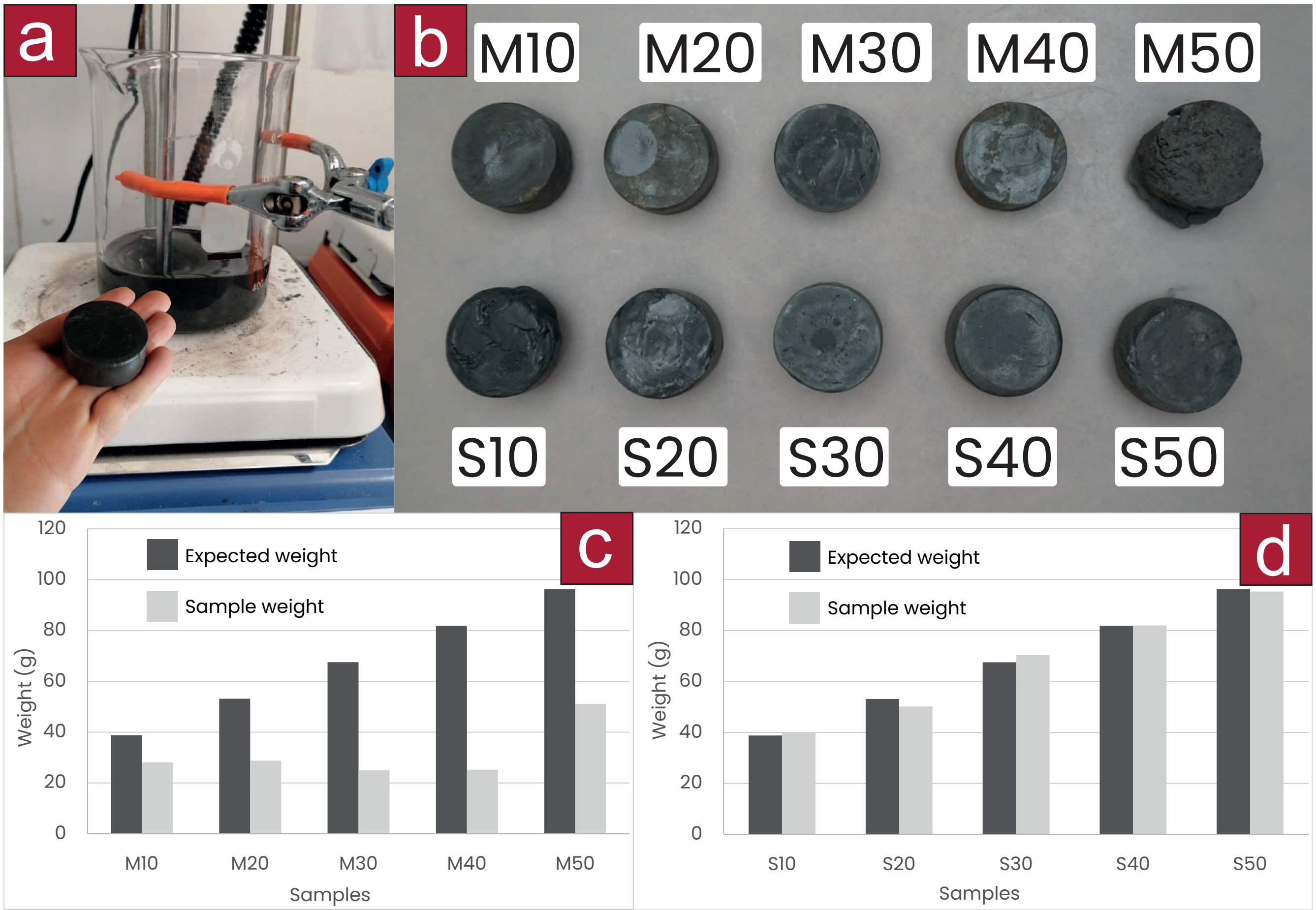

Figure 2. Samples and weight lost using manual and chemical mixing. **a)** Chemical process **b)** Visual aspect of the samples, top are manually mixed, bottom are chemically crafted, left to right with volume proportions of iron from 10% to 50%. **c)** Weight loss with manual mixing. **d)** Weight loss with the solution casting, the chemically created samples are more consistent and the process does not lose components.

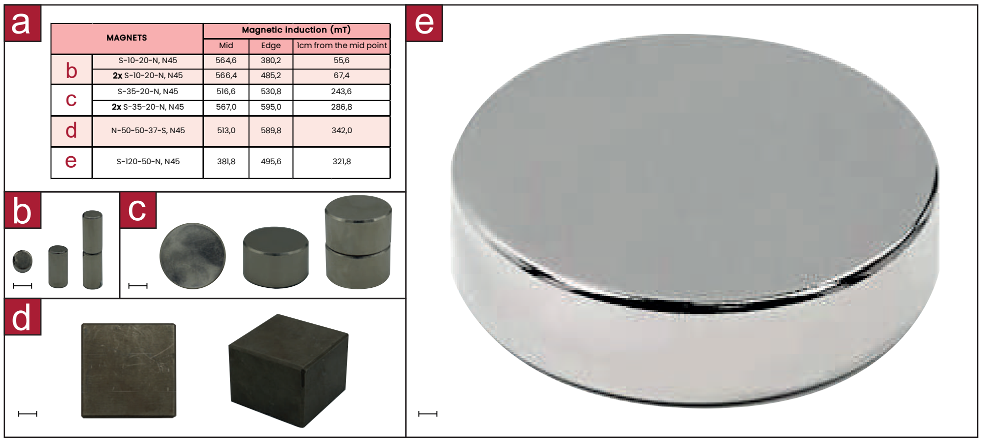

MAGNETS		Magnetic induction (mT)		
		Mid	Edge	1cm from the mid point
b	S-10-20-N, N45	564,6	380,2	55,6
	**2x** S-10-20-N, N45	566,4	485,2	67,4
c	S-35-20-N, N45	516,6	530,8	243,6
	**2x** S-35-20-N, N45	567,0	595,0	286,8
d	N-50-50-37-S, N45	513,0	589,8	342,0
e	S-120-50-N, N45	381,8	495,6	321,8

Figure 3. Magnets employed in the experiments. **a)** Magnetic field strength measured with Teslameter (WT10A) for each magnet on the center of the top surface, 1 cm away from it and on the edge. **b)** Cylinder S-10-20-N, Neodymium N45.**c)** Disc S-35-20-N, Neodymium N45. **d)** Block S-50-50-37-N, Neodymium N45. **e)** Disc S-120-50-N, Neodymium N45. Scale bars are 1cm.

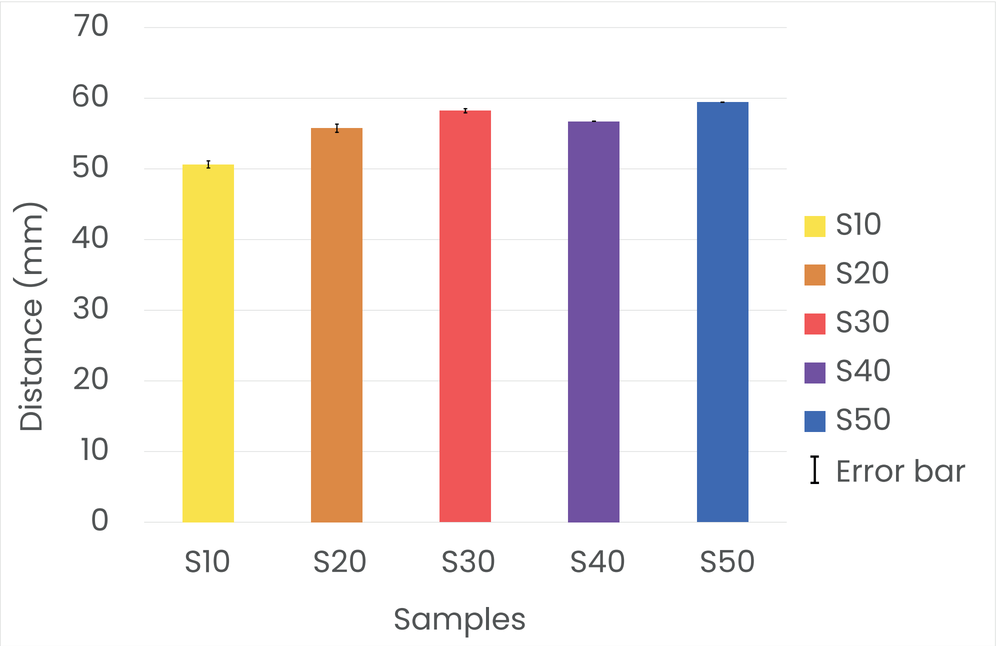

Figure 4. Distance of attraction between a magnet and different samples. For measuring the attraction distance, each sample was placed on a plain surface below the magnetic block (S-50-50-37-N, Neodymium N45) at a distance of 20 cm. Then, the magnet was moved down slowly, until the sample got pulled into the magnet. The attraction distance was calculated subtracting the thickness of the sample.

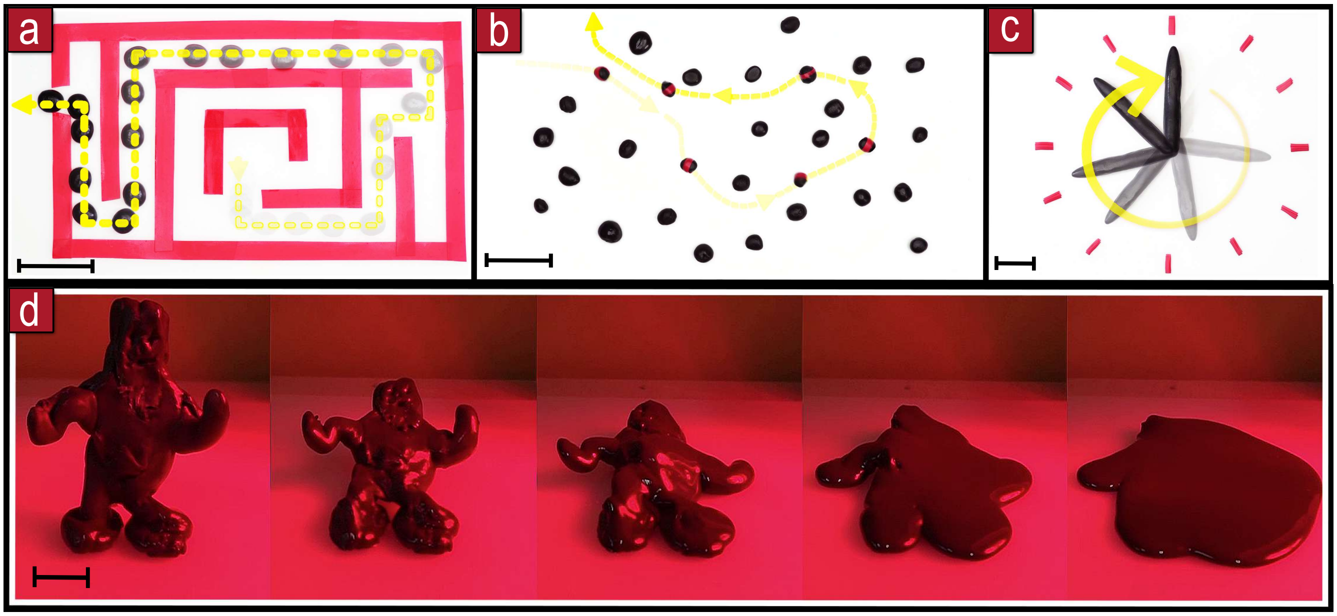

Figure 5. Basic Manipulations that are possible with the material. **a)** Translation. **b)** Translation showing selectivity. **c)** Rotation. **i)** Melting. Scale bars are 3cm.

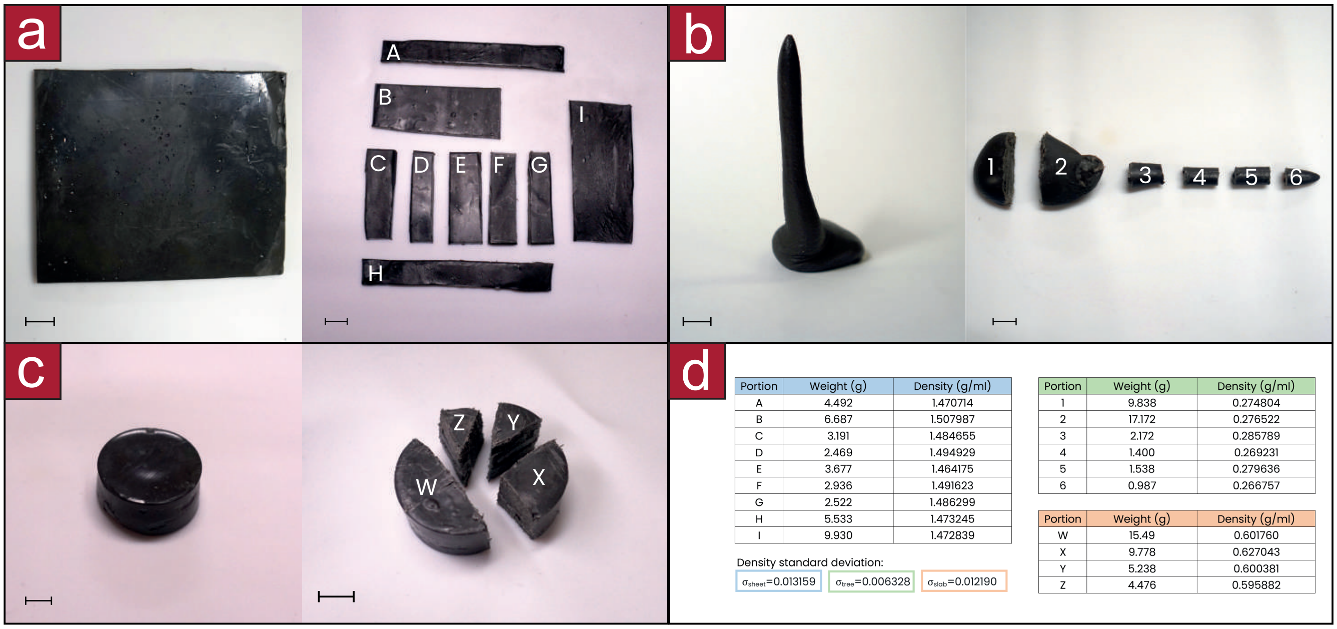

Portion	Weight (g)	Density (g/ml)
A	4.492	1.470714
B	6.687	1.507987
C	3.191	1.484655
D	2.469	1.494929
E	3.677	1.464175
F	2.936	1.491623
G	2.522	1.486299
H	5.533	1.473245
I	9.930	1.472839

Portion	Weight (g)	Density (g/ml)
1	9.838	0.274804
2	17.172	0.276522
3	2.172	0.285789
4	1.400	0.269231
5	1.538	0.279636
6	0.987	0.266757

Portion	Weight (g)	Density (g/ml)
W	15.49	0.601760
X	9.778	0.627043
Y	5.238	0.600381
Z	4.476	0.595882

Figure 6. Density uniformity on the samples: **a)** Sheet with an SD=0.013 g/ml. **b)** Slab magnetically actuated into a spike with an SD=0.006 g/ml. **c)** Cylinder with an SD=0.012 g/ml. **d)** weight and density of each part. The weight was obtained in a microscale TOPINCN of 0.001 g accuracy, the volume was measured by immersion in water in a precision marked pipette. Scale bars are 1 cm.

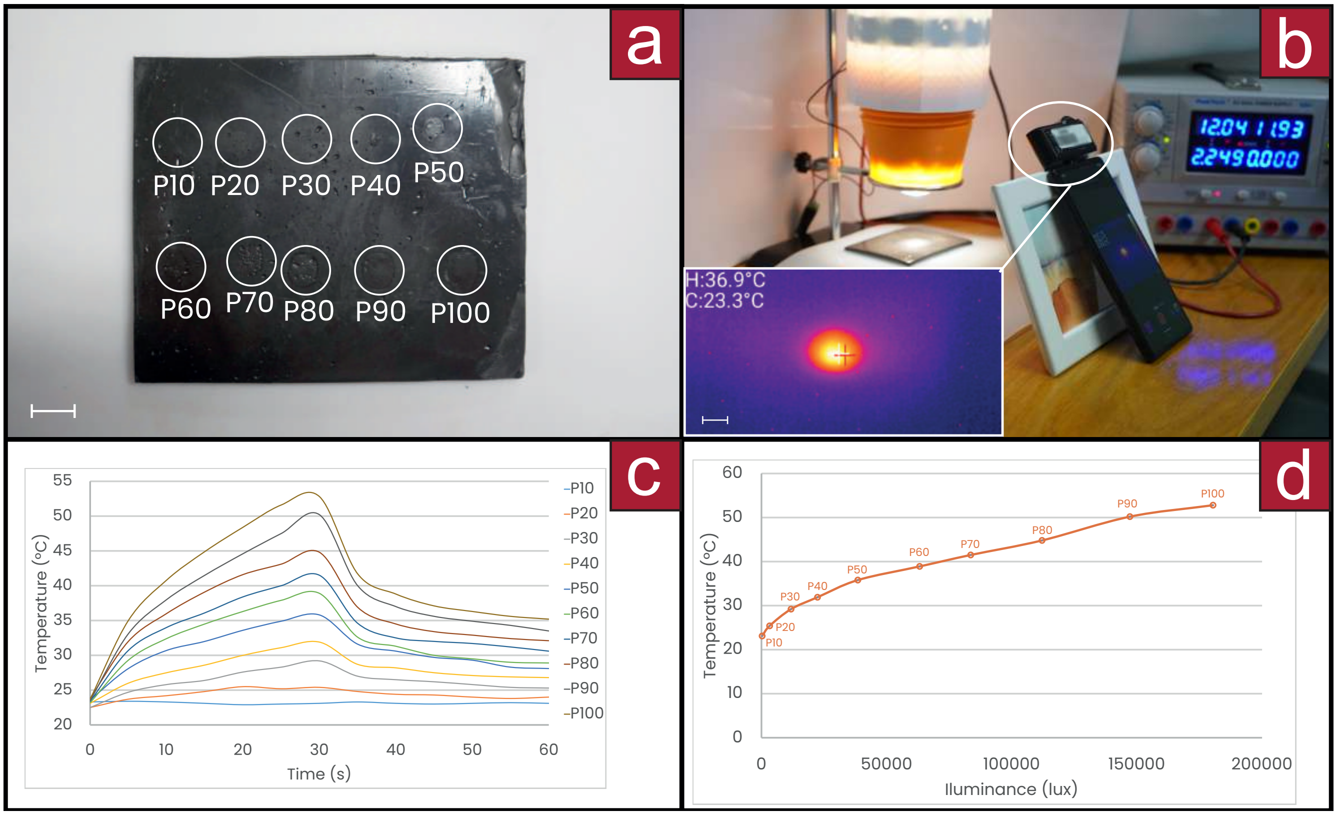

Figure 7. Temperature on the sample for different illumination intensities (P10 to P100). **a)** visual aspect of the sample. **b)** experimental setup. **c)** temperature over time. **d)** temperature at 30 s. for different lux (P10 to P100, 0 to 200 kLux). Scale bars are 1 cm.

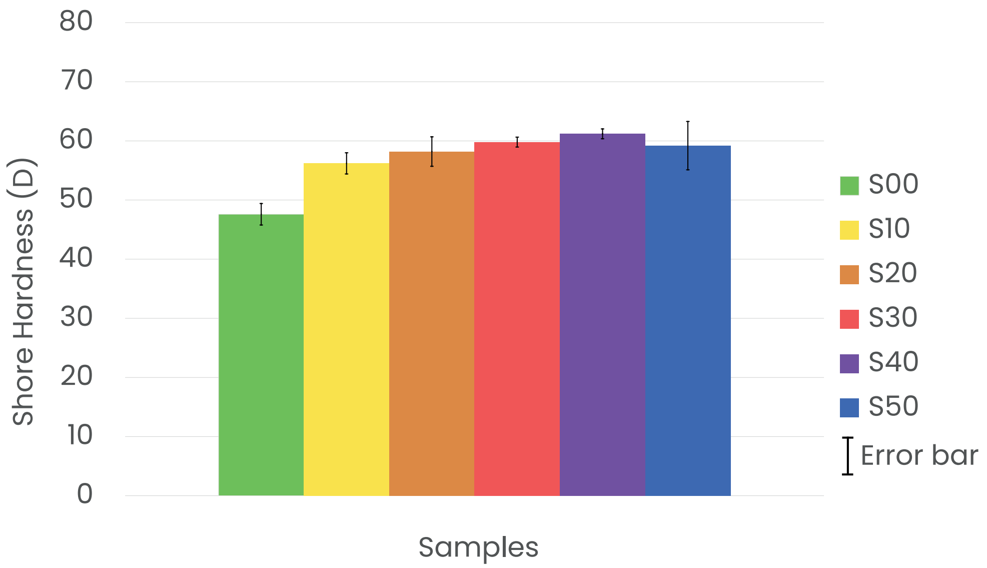

Figure 8. Hardness Shore Test for samples with a different proportion of iron particles (PCL = 0% iron volume, S50 = 50% iron volume). The PLC matrix has a hardness of 47D and it increases as more iron particles are added. However, the S50 sample shows a decrement of hardness due to the lack of adhesion of Fe particles with the PCL matrix. In addition, this sample contain irregularities in its structure and it does not present a homogeneous distribution. This conclusion can be appreciated in the large standard deviation. A Shore type D durometer was employed in this test, it consists of a sharped steel indenter with a diameter of 1.25mm. The measured samples had thicknesses greater than 4mm. Five measurements were taken by sample with a minimum separation of 9mm between indentations. The indentation time was 15s and the essay was performed at a room temperature of 21.5°C and 31 % of relative humidity.

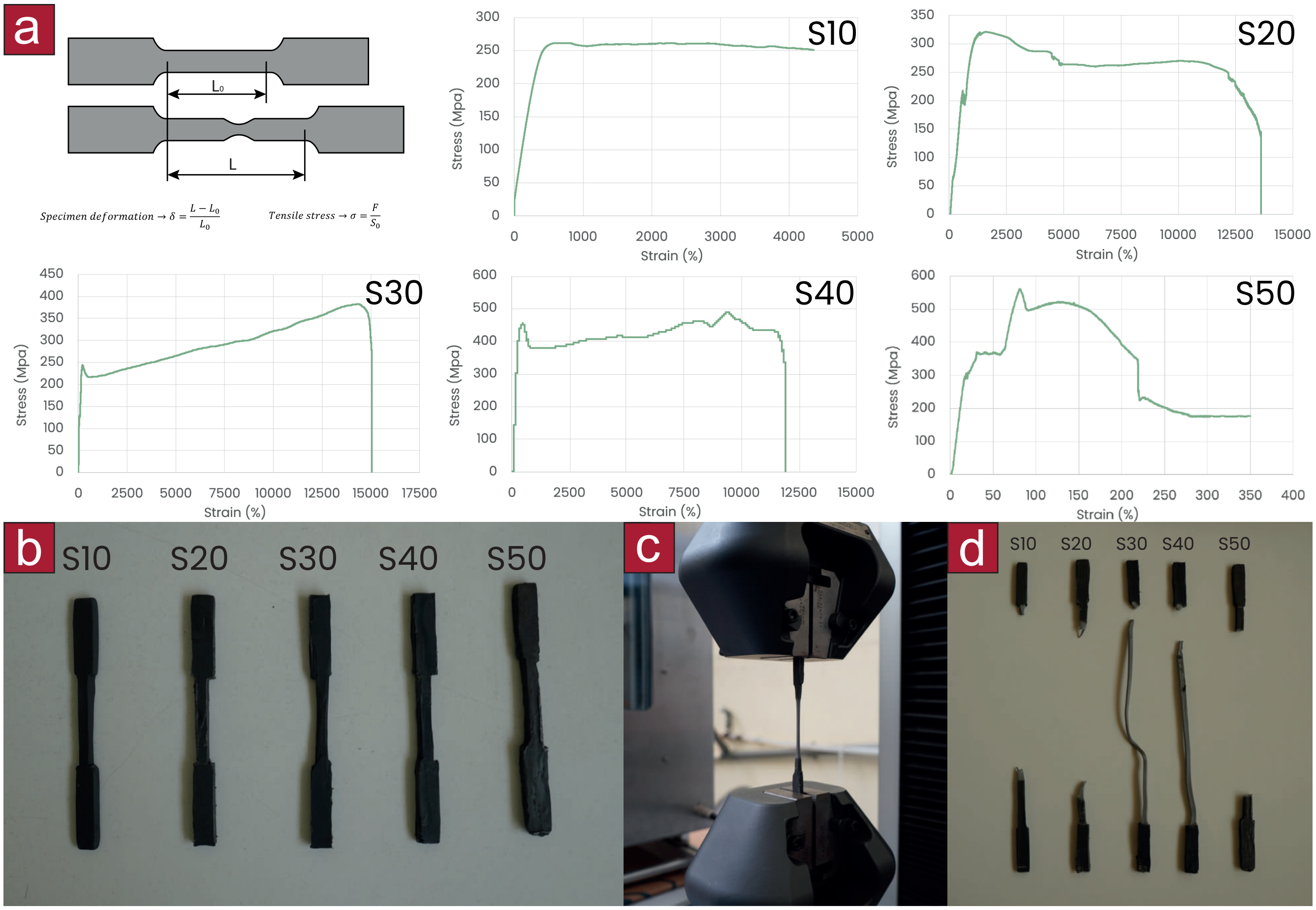

Figure 9. Tensile tests on samples with different proportion of iron volume (S10 = 10% iron volume to S50 = 50% iron volume). **a)** Stress-Strain diagrams for standard tensile test specimens with L_0 = 32mm and 5mm thickness. **b)** Samples before the traction test. **c)** A sample being stretched by the machine. **d)** Samples after the traction test. The required force to break the samples was between 125 and 250 Newtons. The larger the content of iron, the stronger the material. For S30 and S40, it can be observed an elastic extension of the sample. S50 seems to be more brittle.

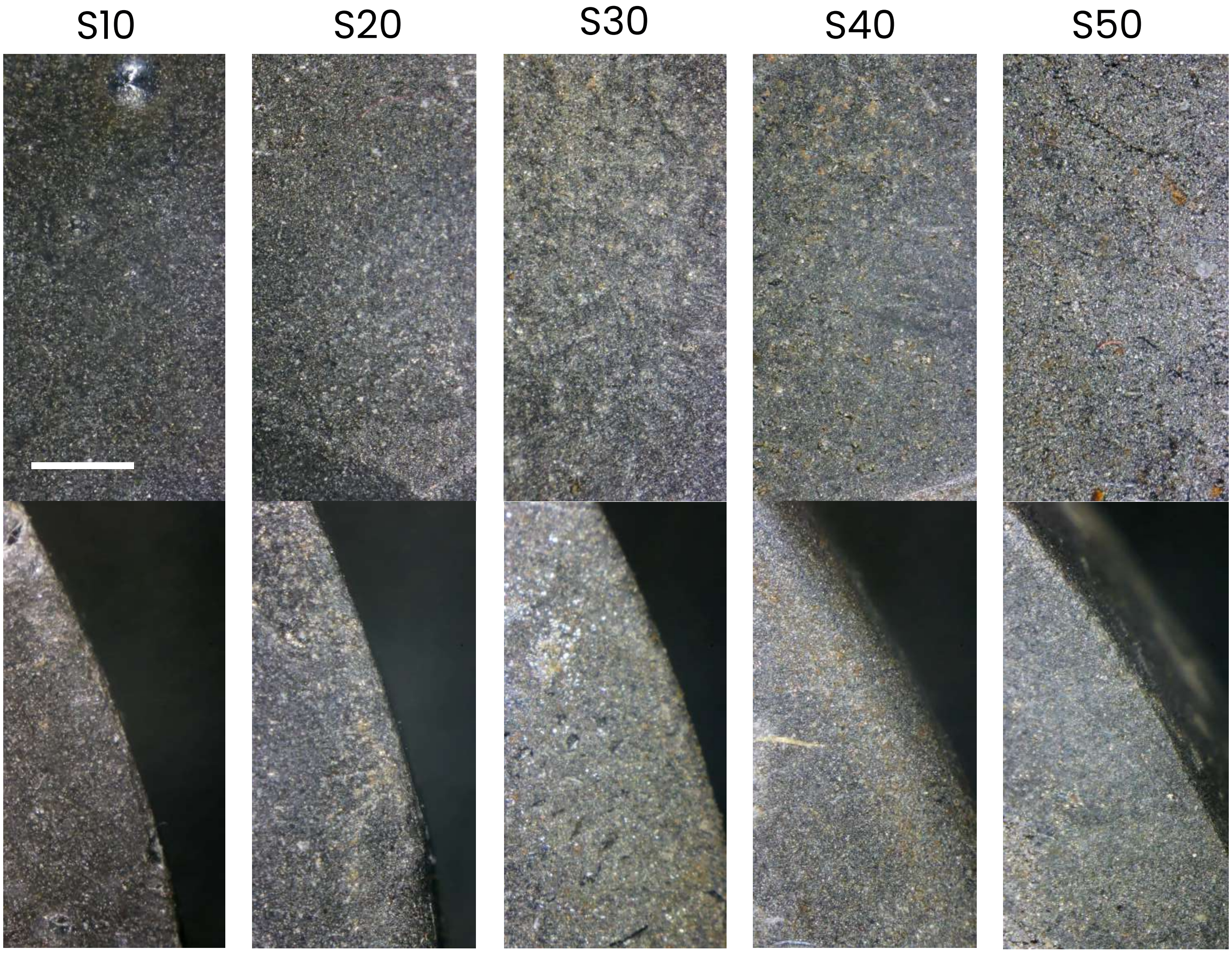

Figure 10. Microscope pictures of different samples at the top of the surface and on the edge of their cylindrical shape. The scale bar is $\approx$ 3mm. The samples were prepared by solution casting and show good homogeneity.

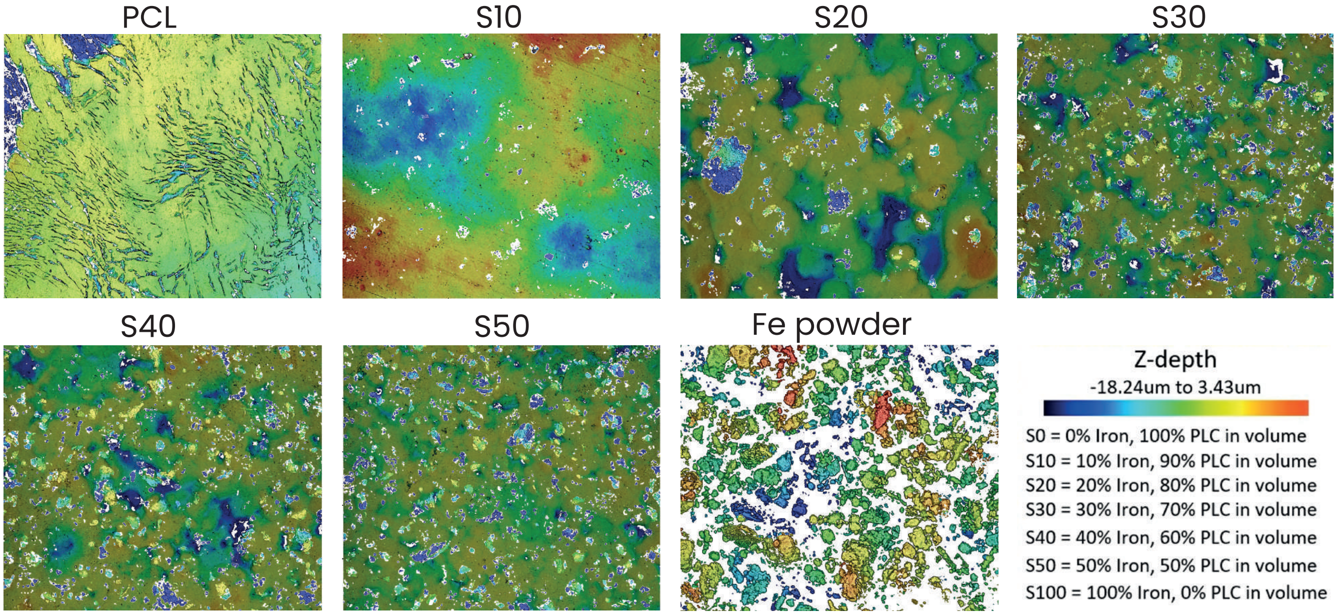

Figure 11. Confocal microscope pictures of different samples at the top of surface. Pure PCL and iron powder were also captured. The increasing amount of iron particles and its homogeneous distribution are seen on the pictures. The pictures are 340x284μm.

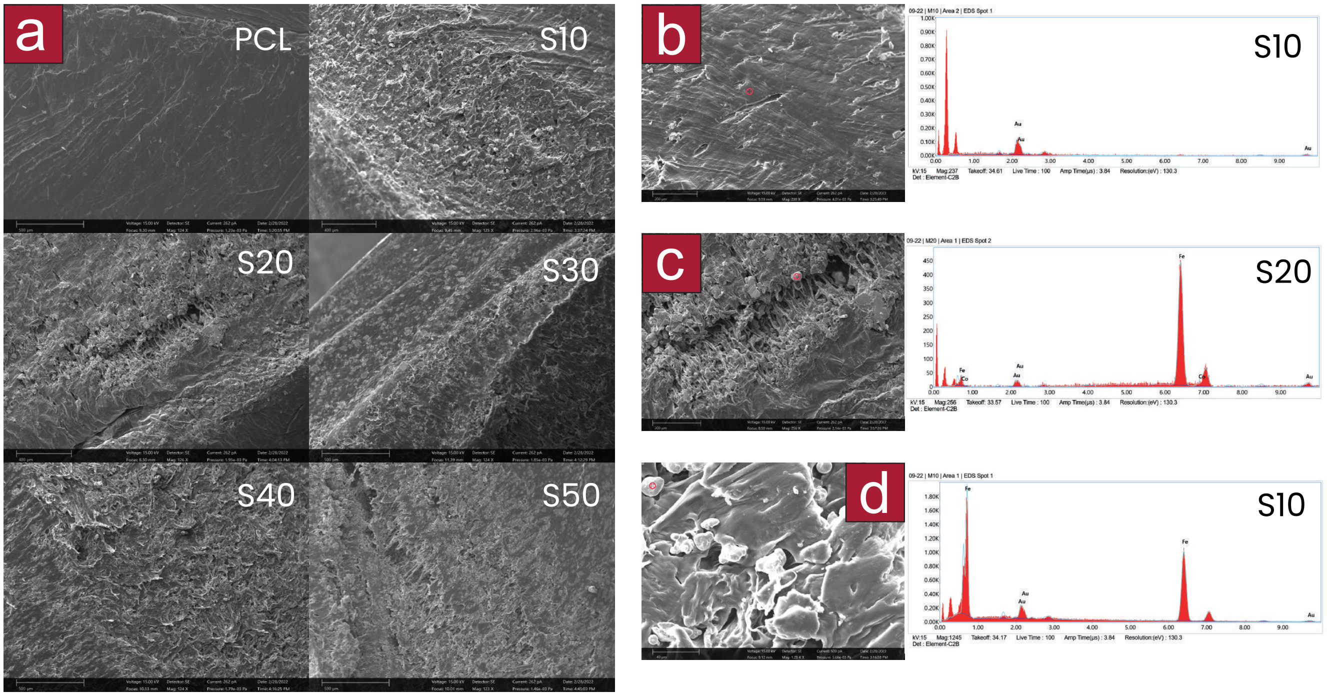

Figure 12. a) Scanning Electron Microscope pictures of different samples. The microscope was operated at 15kV, 262pA and 3mPa. The samples were coated in gold and palladium. The scale bars are 500μm. Electron microscope spectrometry of different parts of the sample with 10% and 20% iron volume. **b)** 237 magnification focused at PCL for S10 sample. **c)** 256 magnification focused on an iron particle. **d)** 1245 magnification focused on an iron particle.
